# Protein Engineering of a Germacrene A Synthase From *Lactuca sativa* and Its Application in High Productivity of Germacrene A in *Escherichia coli*

**DOI:** 10.3389/fpls.2022.932966

**Published:** 2022-08-11

**Authors:** Rong Chen, Yuheng Liu, Shu Chen, Ming Wang, Yao Zhu, Tianyuan Hu, Qiuhui Wei, Xiaopu Yin, Tian Xie

**Affiliations:** ^1^Key Laboratory of Elemene Class Anti-cancer Chinese Medicine of Zhejiang Province, Engineering Laboratory of Development and Application of Traditional Chinese Medicine from Zhejiang Province, School of Pharmacy, Hangzhou Normal University, Hangzhou, China; ^2^School of Public Health, Hangzhou Normal University, Hangzhou, China

**Keywords:** germacrene A, β-elemene, germacrene A synthase, host optimization, site-directed mutagenesis

## Abstract

Germacrene A (GA) is a key intermediate for the synthesis of medicinal active compounds, especially for β-elemene, which is a broad-spectrum anticancer drug. The production of sufficient GA in the microbial platform is vital for the precursors supply of active compounds. In this study, *Escherichia coli* BL21 Star (DE3) was used as the host and cultivated in SBMSN medium, obtaining a highest yield of FPP. The GA synthase from *Lactuca sativa* (LTC2) exhibited the highest level of GA production. Secondly, two residues involved in product release (T410 and T392) were substituted with Ser and Ala, respectively, responsible for relatively higher activities. Next, substitution of selected residues S243 with Asn caused an increase in activity. Furthermore, I364K-T410S and T392A-T410S were created by combination with the beneficial mutation, and they demonstrated dramatically enhanced titers with 1.90-fold and per-cell productivity with 5.44-fold, respectively. Finally, the production titer of GA reached 126.4 mg/L, and the highest productivity was 7.02 mg/L.h by the I364K-T410S mutant in a shake-flask batch culture after fermentation for 18 h. To our knowledge, the productivity of the I364K-T410S mutant is the highest level ever reported. These results highlight a promising method for the industrial production of GA in *E. coli*, and lay a foundation for pathway reconstruction and the production of valuable natural sesquiterpenes.

## Introduction

In nature, sesquiterpenes represent the diverse C15 terpene classes of plant natural products. They have many important pharmacological, physiological, and ecological effects, and are widely used in many fields such as medicine, food, and cosmetics. Germacrene A (GA), which is easily bound to sesquiterpene cyclase, acts as an intermediate for the biosynthesis of various compounds, such as patchoulol and phytoalexins (de Kraker et al., 1998). GA can be converted to germacranes, further producing elemanes, which are an important group of sesquiterpenes widely occurring in nature. Meanwhile, GA can be oxidized into germacrene A carboxylic acid, which is further oxidized to produce the lactone ring, and then functionalized and/or cyclized to the respective guaianolide, eudesmanolide, and germacranolide sesquiterpene lactones (Bouwmeester et al., 2002). GA itself is unstable *in vitro*. GA is particularly susceptible to perform Cope rearrangement toward β-elemene at high temperatures or during freezer storage (de Kraker et al., 1998). Moreover, the step of thermal conversion of GA to ß-elemene was successfully developed by Zhang et al. (2018) which makes the cost of β-elemene only 0.15% of that from plant extraction. β-elemene has been shown to exert activity against a wide range of cancers, including brain, breast, liver, lung, and prostate cancers, as well as drug-resistant tumors. Moreover, Chemo-radiotherapy can be greatly improved by the use of β-elemene, which has few adverse effects on normal tissue cells in clinical trials (Adio, 2009a,b; Wang et al., 2012; Chang et al., 2017). In addition, multidrug-resistant tumor-repopulating cells could be reversed with injections of β-elemene (Zhai et al., 2018, 2019). Because of the important roles of GA both as an intermediate or as an end product, its efficient production in platform microorganisms should be explored using the methods of metabolic engineering and synthetic biology.

Microorganisms have been used for the production of various commercial compounds for a long time. The most common microbial cell factories are the yeast *Saccharomyces cerevisiae*, the bacteria *Escherichia coli*, etc. Since the endogenous mevalonate (MVA) pathway and FPP synthase ERG20 in *S. cerevisiae* could produce farnesyl pyrophosphate (FPP) as the direct precursor for sesquiterpene synthesis (Borodina and Nielsen, 2014), the yeast has been used as a chassis to be engineered for industrial GA production. A yeast platform was established to produce GA with a time-space yield of 2.65 mg/L h in shake flasks (Hu et al., 2017). Subsequently, a time-space yield of 3.44 mg/L h GA was obtained by the best engineered yeast in shake flasks (Zhang et al., 2018). The engineered *S. cerevisiae* expressing a cyanobacterial germacrene a synthase mutant (AvGAS F23W) further improved the production of GA, reaching 4.30 mg/L h in shake-flask batch culture (Zhang et al., 2021). However, no attempts have been made to explore the production of GA in *E. coli*. As the most commonly used host strain in the application of bulk chemical production, *E. coli* has huge advantages such as clear physiological and genetic characteristics, fast growth in minimal salt medium, utilization of various substrates, and easy genetic modification (Liu et al., 2016). Therefore, in this study, we aimed to construct and engineer *E. coli* as cell factories for the highly efficient production of GA.

**SCHEME 1:**
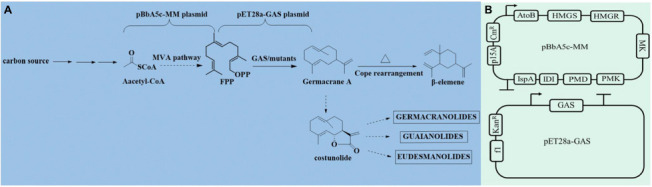
Biosynthesis of germacrene A and its precursors. FPP is produced from acetyl-CoA through the MVA pathway and then form β-elemene or other sesquiterpene lactones **(A)**. The plasmids pBbA5c-MM and pET28a-GASs **(B)**.

*Escherichia coli* generally produces C5 precursors through the endogenous DXP pathway, and then forms FPP, which is used for quinone and cell wall biosynthesis. However, this approach to produce isoprenoid precursors remains ineffective due to regulation mechanisms present in the native host (Yang et al., 2012). Instead, engineering of the heterologous MVA pathway into *E. coli* has been reported to supply sufficient FPP and further improve the productivity of sesquiterpenes (Martin et al., 2003). The evolution of germacrene A synthase (GAS) provides a feasible way to evaluate the catalytic efficiency of the conversion from FPP to GA. In this study, we first introduced the heterologous MVA pathway operons (pMM) in five host *E. coli* hosts, and then screened the optimal culture conditions, to obtain the highest FPP precursor yield. Secondly, screening of GAS from a variety of sources with a high GA yield was performed. Finally, site-directed mutagenesis of the key and selected residues was performed to generate beneficial mutants, and double mutations were combined to further improve the enzymatic activity (Scheme 1). The I364K-T410S was created and demonstrated a dramatically enhanced time-space-yield at 7.02 mg/L.h, which is 1.63∼2.65 fold higher than those previously reported in *S. cerevisiae* on a shake flask fermentation level. Moreover, this study is the first report of GA-production in *E. coli*.

## Materials and Methods

### Strains and Medium

Five *E. coli* strains, including BL21 (DE3), BL21 Star (DE3), BW25113, JM109 (DE3), and BL21 trxB (DE3) were used for gene expression or GA production. Strains were cultivated in SBMSN, LB, and YM9 medium, respectively. The composition of YM9 medium was as follows (L^–1^): yeast extract 2.0 g, Na_2_HPO_4_ 6.0 g, KH_2_PO_4_ 3.0 g, NaCl 0.5 g, NH_4_Cl 1.0 g, MgSO_4_ 1.0 mM, and CaCl_2_ 0.1 mM (Lei et al., 2021). The composition of SBMSN medium was (L^–1^) tryptone 12 g, yeast extract 24.0 g, KH_2_PO_4_ 1.7 g, K_2_HPO_4_ 11.42 g, MgCl_2_⋅6H_2_O 1.0 g, ammonium oxalate 1.42 g and Tween-80 2.0 g (Lei et al., 2021). Antibiotics, 25 mg/L chloramphenicol and 10 mg/L tetracycline were added when necessary.

### Plasmids and Strains Construction

The pBbA5c-MevT–MBIS (abbreviated as pMM) was constructed by the Jay Keasling group and shared from Addgene^1^ (Peralta-Yahya et al., 2011). The pMM, harboring the mevalonate pathway operons and ispA gene, was transformed into *E. coli* BL21 Star (DE3) to generate the engineering strain BL21 Star-MM. The resulting strain could convert acetyl-CoA into FPP. The pMM was respectively transformed into *E. coli* competent cells, including BL21(DE3), BL21 Star (DE3), BW25113, JM109 (DE3), and BL21 trxB (DE3).

To screen the candidate GAS, two literature reported GAS (CbGAS and LTC2) and seven putative GAS genes by BLASTp analysis in the NCBI database were selected, codon-optimized for *E. coli* expression and synthesized by Qinke Biotech Corporation (Nanjing, China). pET28a was used as the expression vector by ligation with these GASs, and the resulting plasmids (pET28a-GAS) were individually transformed into *E. coli* BL21 Star (DE3), generating the expression strains. For *in vivo* yield analysis, each GAS expression vector and pMM were transformed into *E. coli* BL21 Star-MM, generating the co-expressed strains.

### GC Analysis of Farnesol Yield

For *in vivo* farnesol yield analysis, the strains carrying pMM were cultivated in the five different type of medium as previously prepared with 25 μg/mL of chloramphenicol at 37°C. When the cell density at 600 nm reached 0.6–0.8, 0.4 mM IPTG was added to induce protein expression. All flasks were immediately added to 10% (vol/vol) n-dodecane. After 18 h of cultivation, the upper n-dodecane layer was collected, filtered by a 0.22 μm Millipore filter membrane, and diluted with n-hexane at 1: 20 for GC analysis.

GC analysis was performed using an Agilent 2010 series GC system with a flame ionization detector (FID). A GC column HP-5MS (30 m × 250 μm i.d. × 0.25 μm film thickness, Agilent) was employed for separating samples. The system was equilibrated for 2 min at 80°C prior to the subsequent analysis. The temperature program was set as follows: initiation at 80°C, ramp to 140°C at a rate of 40°C per min followed by a 2 min hold, ramp to 260°C at a rate of 40°C per min followed by a 1 min hold. The injector temperatures was set at 260°C while detector temperature was 305°C. Commercial farnesol at the highest purity available (> 99%) was used as control sample (Titan, Shanghai, China). Three biological replicates were performed for collection and GC analysis.

### GC–MS Analysis of Germacrene A Synthase-Catalytic Products

For *in vivo* products analysis, the co-expressing strains, carried pMM and pET28a-GAS, were cultivated in SBMSN medium with 50 μg/mL kanamycin and 25 μg/mL chloramphenicol at 37°C. IPTG induction and product enrichment were performed as described in the farnesol-producing section. Identification of the product was performed by GC–MS as previously described with a slight alternation (Chen et al., 2021). The temperature program was set the same as described above. Commercial β-elemene at the highest purity available (> 99%) was used as control sample (Titan, Shanghai, China).

### Site-Directed Mutagenesis of LTC2

Mutagenesis was generated by PCR using the quick-change site-directed mutagenesis method (Costa et al., 1996). The plasmid pET28a-LTC2 was used as the template and the primers were listed in Table 1. The amplified products were digested with DpnI to remove the original template and then transformed into *E. coli* DH5α competent cells. The positive clones were screened by PCR amplification and sequenced for further verification. The resulting plasmids with mutation were transformed into *E. coli* BL21(DE3) or *E. coli* BL21 Star (DE3)-MM competent cells for further assay.

**TABLE 1:** Plasmids, strains, and primers used in this study.

Plasmids, strains and primers	Description	Source or reference
**Plasmids**		
pET-28a	Integrative plasmid, kan*^r^*	Library stock
pET-28a-LTC2	kan*^r^*,pET-28a-P_*T7*_-LTC2-T_*T7*_	This study
pET-28a-CbGAS	kan*^r^*,pET-28a-P_*T7*_-CbGAS-T_*T7*_	This study
pET-28a-HbSAS	kan*^r^*,pET-28a-P_*T7*_-HbSAS-T_*T7*_	This study
pET-28a-FtSAS	kan*^r^*,pET-28a-P_*T7*_-FtSAS-T_*T7*_	This study
pET-28a-MmGDS	kan*^r^*,pET-28a-P_*T7*_-MmGDS-T_*T7*_	This study
pET-28a-NpGAS	kan*^r^*,pET-28a-P_*T7*_-NpGAS-T_*T7*_	This study
pET-28a-CmGAS	kan*^r^*,pET-28a-P_*T7*_-CmGAS-T_*T7*_	This study
pET-28a-CfGAS	kan*^r^*,pET-28a-P_*T7*_-CfGAS-T_*T7*_	This study
T410S	kan*^r^*,pET-28a-P_*T7*_-LTC2-T410S-T_*T7*_	This study
T410V	kan*^r^*,pET-28a-P_*T7*_-LTC2-T410V-T_*T7*_	This study
T410A	kan*^r^*,pET-28a-P_*T7*_-LTC2-T410A-T_*T7*_	This study
T392A	kan*^r^*,pET-28a-P_*T7*_-LTC2-T392A-T_*T7*_	This study
T392V	kan*^r^*,pET-28a-P_*T7*_-LTC2-T392V-T_*T7*_	This study
T38S	kan*^r^*,pET-28a-P_*T7*_-LTC2-T38S-T_*T7*_	This study
L58V	kan*^r^*,pET-28a-P_*T7*_-LTC2-L58V-T_*T7*_	This study
A229S	kan*^r^*,pET-28a-P_*T7*_-LTC2-A229S-T_*T7*_	This study
S243N	kan*^r^*,pET-28a-P_*T7*_-LTC2-S243N-T_*T7*_	This study
I364K	kan*^r^*,pET-28a-P_*T7*_-LTC2-I364K-T_*T7*_	This study
I492K	kan*^r^*,pET-28a-P_*T7*_-LTC2-I492K-T_*T7*_	This study
S243N-T410S	kan*^r^*,pET-28a-P_*T7*_-LTC2-S243N-T410S-T_*T7*_	This study
I364K-T410S	kan*^r^*,pET-28a-P_*T7*_-LTC2-I364K-T410S-T_*T7*_	This study
T392A-T410S	kan*^r^*,pET-28a-P_*T7*_-LTC2-T392A-T410S-T_*T7*_	This study
T392V-T410S	kan*^r^*,pET-28a-P_*T7*_-LTC2-T392V-T410S-T_*T7*_	This study
NKS	kan*^r^*,pET-28a-P_*T7*_-LTC2-S243N-I364K-T410S-T_*T7*_	This study
KAS	kan*^r^*,pET-28a-P_*T7*_-LTC2-I364K-T392A-T410S-T_*T7*_	This study
pBbA5c-MM	Cm*^r^*,pBbA5c-P_*LacUV5*_-atoB-HMGS(CO)-HMGR(CO)-MK(CO)-PMK(CO)-PMD-idi-ispA-T_*rrnb T1*_	Peralta-Yahya et al., 2011
**Strains**		
*E. coli* BL21(DE3)	F^–^ *ompT hsdS*_*B*_ (r_*B*_^–^ m_*B*_^–^) *gal dcm* (DE3)	Tsingke Co.
*E. coli* BL21 Star(DE3)	F^–^ *ompT hsdS*_*B*_ (r_*B*_^–^ m_*B*_^–^) *gal dcm rne 131*(DE3)	Tsingke Co.
*E. coli* BW25113	F^–^, DE(*ara*D-*ara*B)567, *lac*Z4787(*del*)::*rrn*B-3, LAM-, *rph*-1, DE*(rha*D-*rha*B)568, *hsd*R514	Huayueyang Co.
*E. coli* JM109(DE3)	*end*A1 *rec*A1 *gyr*A96 thi-1 *hsd*R17 (r_*k*_^–^,m_*k*_^+^) *rel*A1 *sup*E44 D (*lac*-*pro*AB) [F’ *tra*D36 *pro*AB *laqI *^q^*ZΔ*M15](DE3)	Weidi Co.
*E. coli* BL21 trxB(DE3)	F^–^ *ompT hsdS*_*B*_ (r_*B*_^–^ m_*B*_^–^) *gal dcm trxB*15::kan(DE3)	Huayueyang Co.
**Primers**		
T410-F	aaaatggtctgattnbwgcgcatataatg	This study
T410-R	wvnaatcagaccattttttcatattccgg	This study
T392A/V-F	agaagcagaatgggynaatagcgttatg	This study
T392A/V-R	nrcccattctgcttcttccagataaccacg	This study
T38S-F	gatcgttttctgagctttagcctggataat	This study
T38S-R	ctaaagctcagaaaacgatcaccccaaa	This study
L58V-F	aagcaccgaaagaagaagtgcgtcgtct	This study
L58V-R	cttcttctttcggtgcttccattgcttttgcat	This study
A229S-F	ttatagcgaagaatgtagcacccatgaat	This study
A229S-R	gctacattcttcgctataattgctaaaatac	This study
S243N-F	ctggcaaaactgcattttaactatctggaact	This study
S243N-R	ttaaaatgcagttttgccagtttcagcagac	This study
I364K-F	aaaaacagctggcaaaagaaggtcgtg	This study
I364K-R	ttttgccagctgtttttccagttctgcat	This study
I492K-F	gcaattgatgaactgaaaaaaatgattgaaa	This study
I492K-R	ttttttcagttcatcaattgcttctttttcgc	This study

### Protein Preparation and *in vitro* Enzyme Assay

The *E. coli* BL21(DE3) strains carrying pET28a-LTC2 or mutant sites were grown in LB medium with 50 μg/mL kanamycin at 37°C until the cell density at 600 nm reached 0.6, followed by induction with 0.1 mM IPTG overnight to produce N-terminal His_6_-tagged recombinant proteins. The expression level of His-fusion proteins was detected by SDS–PAGE. The protein concentration was quantified using the Bradford method. Activity assays of LTC2 and mutants were performed in a volume of 1 mL containing 20 μg of protein, 0.1 M Tris-HCl (7.0), 3 mM MgCl_2_, 0.1 M DTT, 0.15 M 50% glycerin, and 1 μL *E.E*-FPP (Sigma–Aldrich) (Bennett et al., 2002). The reaction mixture was conducted at 37°C for 1 h, and the products were collected by headspace solid phase micro extraction (SPME) fiber. After 60 min of sampling, the fiber was removed and immediately transferred to the injection port of the GC. GC analysis was performed as described in the farnesol-producing section with β-elemene as the standard.

### Protein Modeling and Docking Analysis

The three-dimensional homology model of LTC2 was generated on the Swiss-Model server.^2^ The X-ray structure of tobacco 5-epi-aristolochene synthase (PDB ID: 5EAU) was chosen as the best template (Starks et al., 1997). Docking of the FPP ligand into the model of the LTC2 active site cavity was performed *via* setting the parameters of “copy ligand from templates” by Discovery Studio 2020 software (Accelrys, San Diego, CA, United States). The GA was docked into the LTC2 structure by AutoDock Vina program (Trott and Olson, 2010). The generated model was geometrically refined by the Princeton TIGRESS 2.0 (Khoury et al., 2017). The final model was evaluated using Procheck program, and the lowest energy conformational model was chosen for docking studies (Chen et al., 2016).

## Results

### Effect of Strains and Medium on Farnesol Yield

**FIGURE 1:**
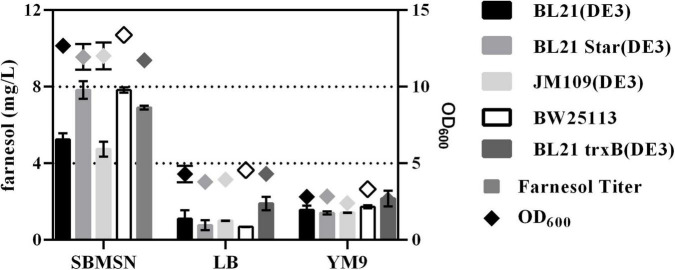
Effect of medium and strains on farnesol yield and OD_600_ in *E. coli* hosts carrying pMM.

The expression level of heterologous gene was affected by many factors such as host strains, cell growth, inducer concentration, etc. (Yin et al., 2015). It is an effective way to improve exogenous gene expression by optimizing the host and culture medium. Because FPP can be spontaneously hydrolyzed into farnesol by endogenous phosphatase or pyrophosphatase (Wang et al., 2016), the level of farnesol was used to evaluate the ability of FPP production in *E. coli*. As shown in Figure 1, five different host strains carrying pMM could produce more farnesol in SBMSN medium than those in LB and YM9 medium. For example, the engineered strain BL21(DE3)-MM in SBMSN medium produced 4.88-fold and 3.34-fold farnesol than those in LB and YM9 medium, respectively. Farnesol exhibited higher levels in BL21(DE3) Star, BW25113, and BL21 trxB (DE3) strains grown in SBMSN medium. Overall, The BL21Star (DE3) strain showed the highest per-cell productivity of farnesol yield (1.55 mg/g. DCW). *E. coli* BL21 Star (DE3) is characterized by containing a mutated rne131 gene encoding ribonuclease E (RNaseE), which can enhance the stability of messenger RNA, which in turn elevates the expression level of target proteins (Lee et al., 2020). Hence, the *E. coli* BL21 Star (DE3) carrying the plasmid pMM was used as a chassis and cultivated in SBMSN medium to evaluate the yield of GA.

### Screening of Germacrene A Synthase

**FIGURE 2:**
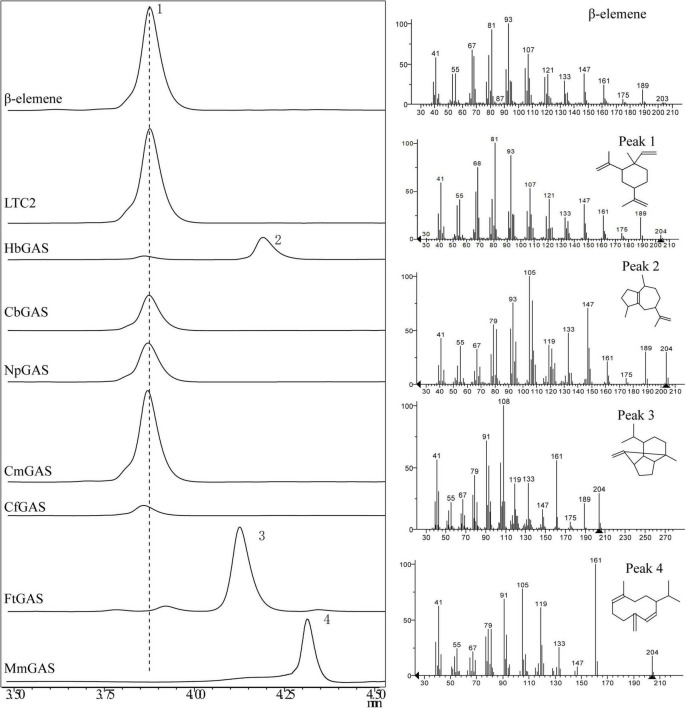
GC-MS analysis of products in *E. coli* BL21Star (DE3) pMM- GASs. Peaks 1, β-elemene; 2, guaiene; 3, sativene; 4, germacrene D.

**FIGURE 3:**
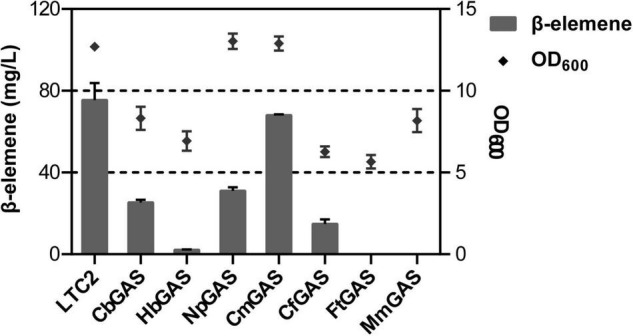
β-elemene titer and OD_600_ analysis of *E. coli* BL21Star (DE3) pMM- GASs.

For selecting an efficient GAS, two literature reported GASs (LTC2 and CbGAS) were overexpressed in *E. coli* BL21 Star (DE3)-MM engineered strain. LTC2 was identified from *Lactuca sativa* while CbGAS was identified from the bacterium *Nostoc* sp. PCC 7120 (Bennett et al., 2002; Agger et al., 2008). To mine candidate GAS, BLSATP was performed with CbGAS in the Genbank database. Finally, six genes with 37–92% amino-acid sequence similarities were selected for yield analysis, including *HbGAS* (*Hassallia byssoidea*), *MmGAS* (*Methyloglobulus morosus*), *FtGAS* (*Fischerella thermalis*), *NpGAS* (*Nostoc parmelioides*), *CmGAS* (*Calothrix membranacea*), and *CfGAS* (*Calothrix* sp. FI2-JRJ7). GC–MS confirmed that LTC2, CbGAS, NpGAS, CmGAS, and CfGAS could produce β-elemene as the sole product in the engineered *E. coli*. The main product of HbGAS was guaiene, concomitant with a small amount of β-elemene. FtGAS and MmGAS generated other sesquiterpenes, sativene and germacrene D, respectively (Figure 2). Among them, LTC2 obtained the highest yield of β-elemene with 75.45 mg/L after 18 h of fermentation and growth (OD_600_ = 12.1) (Figure 3). However, LTC2 showed no β-elemene-producing capability in yeast cells (Zhang et al., 2021). We inferred that the mildly acidic intracellular environment of yeast cells led to low activity for LTC2, consequently, the effect of pH on LTC2 was performed. At the pH conditions (about 6.8) in yeast cytosol, LTC2 maintained only 40% relative activity. The optimal pH of LTC2 was 7.5 (Supplementary Figure 1). SDS–PAGE confirmed that these bacterial origin GASs were largely expressed as inclusion bodies in *E. coli*, resulting in a low content of soluble recombinant enzymes and eventually a low β-elemene yield. LTC2 could be expressed as a soluble protein with a few amount of inclusion in the whole cell lysates (Supplementary Figure 2). Hence, LTC2 was selected for further research.

### T410S and T392A Mutants Improve the Germacrene A Yield

**FIGURE 4:**
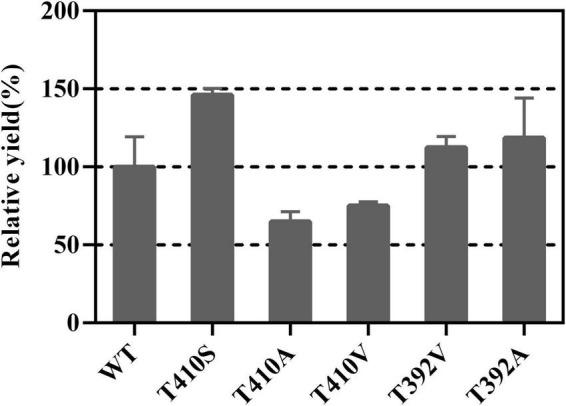
The relative yields of T410 and T392 mutants compared with WT.

First, we sorted some mutation sites with enhancing activity in sesquiterpene synthase family. For example, the threonine residue at the 399 position of Artemisia annua amorpha-4,11-diene synthase (AaADS) was replaced with serine (T399S), resulting in a catalytic efficiency improvement (Li et al., 2013). The leucine residue at the 381 position of *A. annua* α-bisabolol synthase (AaBOS) was substituted with alanine (L381A), exhibiting an approximate twofold increase in the production of γ-humulene compared with the wide type (WT) (Li et al., 2013). Corresponding to T399 of AaADS and L381 of AaBOS, T410 and T392 of LTC2 were chosen to be replaced with Ser and Ala, respectively. Ala and Val with a small side chain may lead to the easy release of product. Hence, T410A, T410V, T392A, and T392V were constructed. All mutants of T410 and T392 showed primarily soluble expression in *E. coli* BL21 Star (DE3) (Supplementary Figure 3). *In vitro* enzymatic analysis verified that the relative yield of T410S raised to 146% compared with the WT, while the T410A and T410V mutants partially lost their activities. The T392A and T392V had slightly increasing activities toward FPP (Figure 4). Overall, the T410S and T392A exhibited relatively increasing activities compared with the WT.

### Effects of Selected Mutants on Germacrene A Yield

**FIGURE 5:**
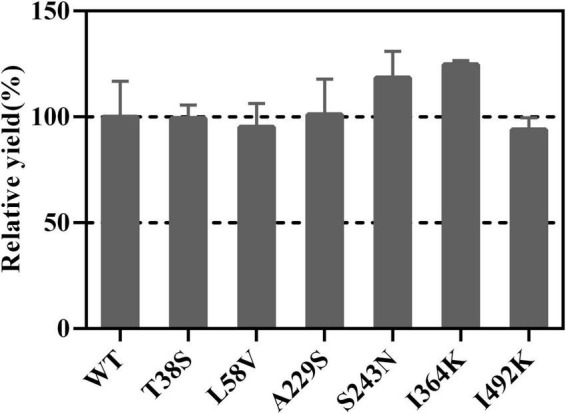
The relative yields of selected mutants compared with WT.

Mutation of residues involved in the active center or conserved sites was disregarded, which possibly led to enzyme inactivation, product profile alteration and catalytic efficiency lowering (Bibi et al., 2020). Plasticity residues in or around the active center may increase the product diversity or improve the catalytic efficiency (Yoshikuni et al., 2006; O’Maille et al., 2008). However, the mutation of GAS exists some difficulties, such as the complex catalytic mechanisms, the lack of appropriate crystal structure, and an effective high-throughput screening approach. Hence, those residues, which are conserved but non-critical for catalysis in GAS, were chosen for mutation. Sequence alignment was performed using LTC2 and literature-reported plant-derived GASs, including BsGAS1, HaGAS1, HaGAS2, CiGASsh, CiGASlo, and ToGAS2 (Bouwmeester et al., 2002; Göpfert et al., 2009; Huber et al., 2016; Nguyen et al., 2016). The sequence alignment revealed that six residues were identical at the corresponding positions in these GASs but distinct in LTC2 (Table 2). Therefore, the T38S, L58V, A229S, S243N, I364K, and I492K mutants were taken into consideration for mutation. All mutants were expressed solubly in *E. coli* (Supplementary Figure 4) and retained activities toward FPP *in vitro*. Among them, I364K and S223N were slightly increased in relative yield compared with WT which were chosen for further mutation (Figure 5).

**TABLE 2:** The selected residue positions in LTC2 and plant-derived GASs.

		Amino acid position					
Species	Name	38	58	229	243	364	492
*Lactuca sativa*	LTC2	T	L	A	S	I	I
*Barnadesia spinosa*	BsGAS1	S	V	S	N	K	K
*Helianthus annuus* L.	HaGAS1	S	V	S	N	K	K
	HaGAS2	S	V	S	N	K	K
*Cichorium intybus*	CiGASsh	S	V	S	N	K	K
	CiGASlo	S	V	S	N	K	K
*Taraxacum officinale*	ToGAS2	S	V	S	N	K	K

### Combination Analysis of Mutation Sites

**FIGURE 6:**
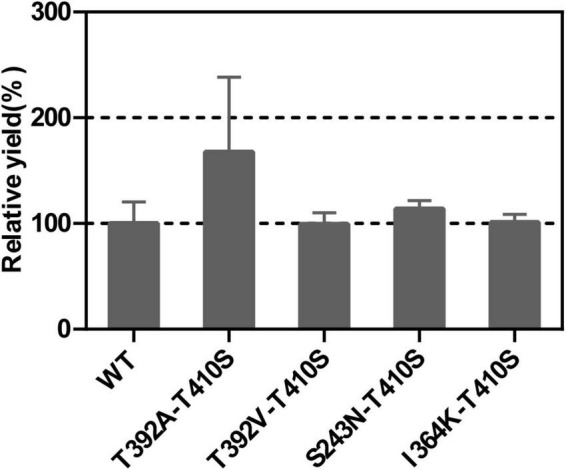
The relative yields of LTC2 double mutants compared with WT.

It’s an effective way to improve enzyme performance by combinant mutations, which often produce an additive effect (Wang et al., 2015). Therefore, we used the T410S mutant as the template and conducted site-directed mutagenesis to generate double mutants that further improved the activity of LTC2. The *E. coli* BL21 Star (DE3) carrying mutant plasmids were expressed solubly in *E. coli* (Supplementary Figure 5). *In vitro* enzymatic analysis showed that the T392A-T410S mutant demonstrated a significantly increased relative yield while others did not compared with the WT (Figure 6).

### *In vivo* Evaluation of Mutants for Germacrene A Production

**FIGURE 7:**
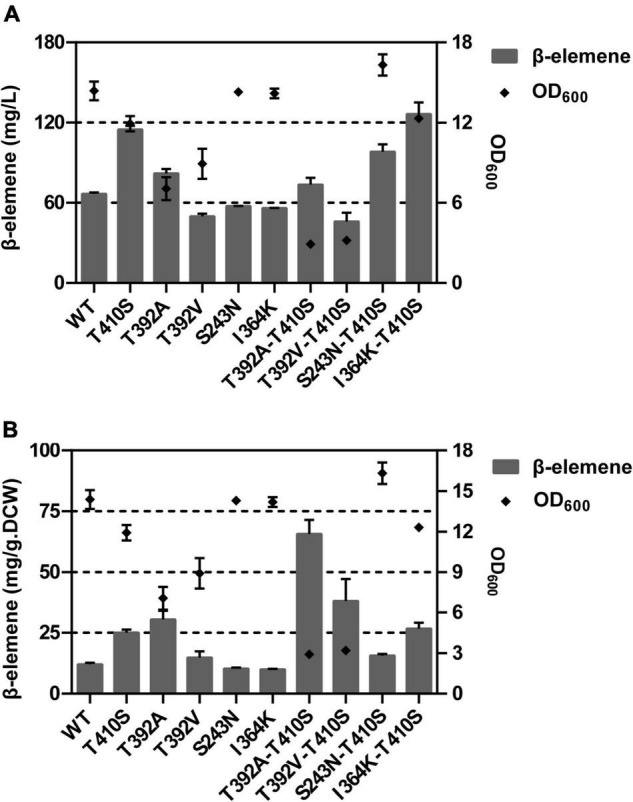
The titer **(A)** and per-cell productivity **(B)** of β-elemene in *E. coli* BL21 Star (DE3) pMM – LTC2 mutants.

Next, the enrichment in n-dodecane and quantitative analysis of volatile GA by GC after 18 h cultivation was performed for all mutants. The results of the combinatorial mutagenesis showed that the GA yield of I364K-T410S was 126.4 mg/L, while that of WT was 66.7 mg/L. The I364K-T410S was 1.90 time in the amount of β-elemene production, compared with WT. We found that these engineered strains after 18 h of induced culture exhibited a large growth difference, so the value of β-elemene yield was also reported as micrograms per gram of dried weight (mg/g DCW). The T392A-T410S mutant exhibited the highest yield of β-elemene with 65.7 mg/g DCW, a 5.44 fold of the WT (Figure 7). These results demonstrated the synergistic effects of beneficial mutations.

## Discussion

**FIGURE 8:**
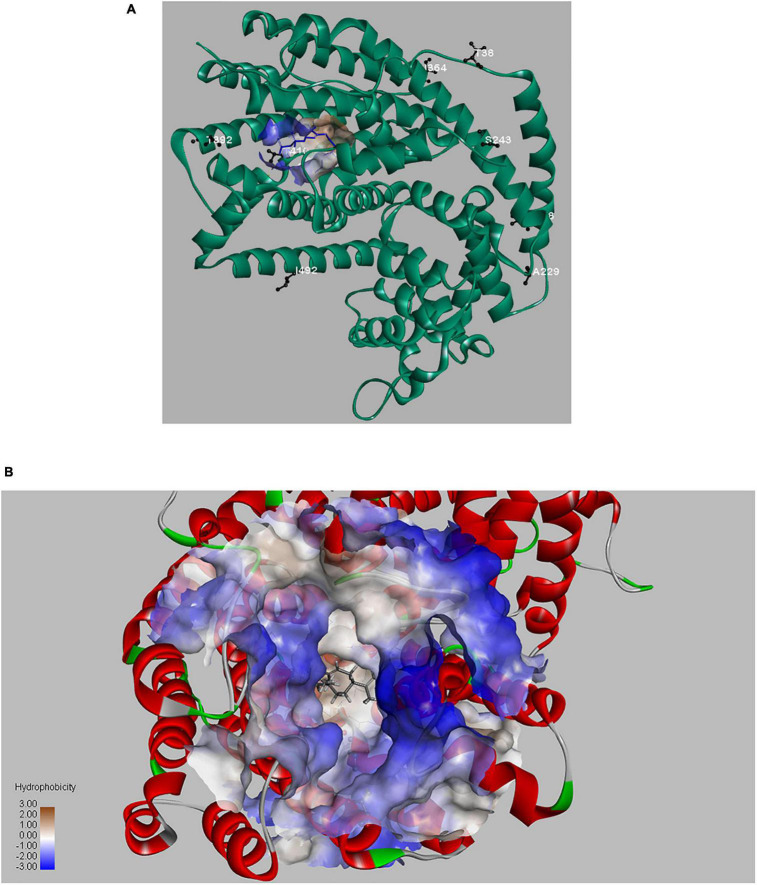
The overview structure of LTC2 and mutant residues **(A)**; LTC2-FPP interaction and active channel **(B)**.

In recent years, many protein engineering approaches have been developed, such as rational design, directed evolution, *de novo* design, computational-aided approaches etc., which have enabled novel proteins with improved properties, including better enzymatic properties, better stability, improved catalytic activity or advanced applications (Sinha and Shukla, 2019). Because of the complexity of enzymatic catalysis, especially the terpene synthase (TPS), a combination of protein engineering approaches may be advantageous (Yoshikuni et al., 2006; Fang et al., 2017). In our research, improved LTC2 mutants were obtained by protein engineering approach. To better understand the positive effects of these selected sites on LTC2 function, we performed structural simulation analysis (Figure 8A). The three-dimensional homology model of LTC2 was obtained by comparative modeling using the tobacco 5-epi-aristolochene synthase (TEAS, PDB ID: 5EAU). LTC2 shared 40.6% sequence-similarity with TESA. The obtained models of LTC2 was structurally similar to TEAS, comprising two structural domains: the C-terminal catalytic domain and the N-terminal domain with unknown function (Starks et al., 1997; Rudolf and Chang, 2020). These highly conserved motifs of DDXXD, RXR, and NSE/DTE of terpene synthase family were found in its primary sequence. As reported in TPS, the DDXXD motif binds with metal Mg^2+^, playing a role in promoting multiple orientations of the substrate alkyl chain. The RXR motif, located in the entrance of the enzymatic pocket, acts in protecting the hydrophobic active center from water molecules invading (Liu et al., 2018). The catalytic mechanism of terpene synthase is complicated and may include the following steps: enzyme-substrate binding and folding, the generation and stabilization of high-energy carbocations, the acidic/basic catalysis to form specific skeletons or metabolites, as well as product release (Li et al., 2013). Moreover, the last step of product release is often a rate-limiting step (Rising et al., 2000). Deprotonation is a general way to speed up the formation of olefinic products for most terpene synthases (TPS) (Fang et al., 2017). In this study, the substitution T of 410 with S in LTC2 led to a 46% increase in catalytic activity and a 72% increase of GA yield. The serine residue is structurally similar to the threonine residue. Both have a polar side chain and a hydroxy group, but the serine is smaller than the threonine in structure. Hence, the change from threonine to serine in LTC2 may act a favored effect in the last reaction step-deprotonation process. Moreover, the serine has a more polar side chain (–CH_2_OH) than the threonine (–CH(OH)CH_3_), resulting in a more hydrophilic pocket environment to accelerate dissociation rate of hydrophobic product (Li et al., 2013). As we anticipated, the threonine at 392 of LTC2 was located on the rim of active pocket and may affect the conformation of the FPP entry or GA exit channel. The replacement of T392 with alanine may decrease the steric hindrance on the rim of FPP entrance or GA exit of the pocket (Figure 8B). Thus, T410S and T392A of LTC2 exerted favorable effects (Li et al., 2013).

Usually, functionally important amino acids are evolutionarily conserved in the primary structure. However, mutation of these key residues in or around the active center would result in inactivation of enzyme (Zabala et al., 2012; Bibi et al., 2020). Therefore, substitution of residues that are conserved but non-critical to catalysis may exert a beneficial effect. Based on multiple sequence alignment of LTC2 and other GA-produced GASs, six residues were found to be identical at the corresponding positions in literature reported GASs but distinct in LTC2. Ultimately, only purified S243N and I364K mutants exerted slightly favorable effects *in vitro*. As shown in Figure 8A, T38, L58, A229, and I492 were located in the N-terminal α-barrel domain and far from the active center, leading to no effect on the enzymatic activity. S243N and I364K were predicted to be located in the C-terminal α-helix domain but still far from the enzyme active center (Figure 8A). However, their elevated levels were limited *in vitro*, even with a minor decrease in *E. coli* BL21 Star (DE3)-MM strains. Furthermore, a combination analysis of beneficial mutants was performed. As a result, the T392A-T410S and I364K-T410S presented increased productivities and titers of GA, respectively. Actually, the triple mutants of KAS(I364K-T293A-T410S) and NKS(S264N-I364K-T410S) were also constructed but showed unfavorable titers of GA, compared with their corresponding double mutants (Supplementary Figure 6).

The combined analysis of GA titer and OD_600_ revealed that these strains expressed T392A alone or together with T410S showed lower biomass. Therefore, the T392A-T410S exhibited the highest per-cell productivity of 65.8 mg/g DCW. Whereas, I364K-T410 showed the highest product yield of 126.0 mg/L after 18 h of fermentation and a similar biomass compared with WT. Overall, the per-cell productivity of the strain carrying T392A-T410S exhibited the highest level of GA, probably results of improvement in thermo-stability or catalytic efficiency that need to be further verified by experiments. Furthermore, the crystal structures of WT and mutant enzymes alone or with FPP substrate/GA product should be performed to explain the mechanism.

Germacrene A is a key intermediate for the synthesis of various active compounds, especially for β-elemene, a broad-spectrum anticancer drug. The thermal conversion of GA to β-elemene undergoes very easily. At present, the only way to obtain elemene is extraction from the ginger plant *Curcuma wenyujin*. However, these factors, such as low content, complex extraction procedure and high extraction cost, have largely restricted its practical applications. Consequently, we used host/medium optimization and protein engineering approaches to generate an engineered *E. coli* BL21 Star (DE3)-MM-LTC2_*mutant*_ strain to yield GA.

First, to achieve a higher FPP production, we developed the extraction fermentation with n-dodecane as overlay, and optimized cultivation for 18 h and IPTG concentration at 0.4 mM. Under this condition, the strain BL21(DE3) Star carrying pMM produced the highest titer of about 7.83 mg/L FPP in SBMSN medium. On the contrary, the engineering strain BL21(DE3) Star-pMM had a lower biomass and produced a low titer of FPP in LB or YM9 medium. An integrated consideration of these factors, the strain BL21(DE3) Star-pMM was used as the best host and cultivated in SBMSN medium, aiming to supply sufficient precursors. Finally, co-expressed with GAS mutant, the strain BL21(DE3) Star-pMM-GAS_*I*364*K–T*410*S*_ mutant exhibited a time-space-yield of 7.02 mg/L.h, which is 1.63∼2.65 fold of those previously reported in *S. cerevisiae* on a shake flask fermentation level (Table 3). Although the final titer of the engineered strain was lower than that of *S. cerevisiae*, more biotechnologies and engineering strategies should be developed for increasing GA production. Usually, the successful implementation of new bioprocesses requires the maximization of product yield, titer, and productivity. However, these performance indicators cannot be maximized simultaneously due to the inherent trade-off between the biomass and the target product (Klamt et al., 2018). Some optimization strategies have been successfully established. For example, high cell density fermentation could increase biomass reaching an OD_600_ of 160–180, using a chemically defined fermentation medium for *E. coli* growth (Yang et al., 2020; Perl et al., 2022). Alternatively, two-stage fermentation, composed of cell growth and production, makes it easy to achieve the improved productivity of biotechnological production processes (Klamt et al., 2018). It can be expected that the yield of GA could be further improved to a very high level by these optimizing strategies.

**TABLE 3:** Comparison of data for the production of β-elemene in engineered strains.

Chassis	GAS	Titer (mg/L)	Time (h)	Productivity (mg/L h)	References
*Saccharomyces cerevisiae*	AvGAS-F23V	309.8	72	4.30	Zhang et al., 2021
*Saccharomyces cerevisiae*	LTC2	190.7	72	2.65	Hu et al., 2017
*Saccharomyces cerevisiae*	LTC2	469	144	3.26	Zhang et al., 2018
*E. coli*	LTC2-I364K-T410S	126.4	18	7.02	This research

## Data Availability Statement

The original contributions presented in the study are included in the article/Supplementary Material, further inquiries can be directed to the corresponding authors.

## Author Contributions

RC: conceptualization, methodology, writing – original draft, and writing – review and editing. YL: methodology, formal analysis, and data curation. SC, MW, and YZ: methodology. TH: writing – original draft. QW: formal analysis. XY: conceptualization, supervision, and funding acquisition. TX: conceptualization, supervision, and project administration. All authors contributed to the article and approved the submitted version.

## Conflict of Interest

The authors declare that the research was conducted in the absence of any commercial or financial relationships that could be construed as a potential conflict of interest.

## Publisher’s Note

All claims expressed in this article are solely those of the authors and do not necessarily represent those of their affiliated organizations, or those of the publisher, the editors and the reviewers. Any product that may be evaluated in this article, or claim that may be made by its manufacturer, is not guaranteed or endorsed by the publisher.

## Footnotes

1
www.addgene.org

2
http://www.expasy.org
