# Supplementary material for: Protein Engineering of a Germacrene A Synthase From Lactuca sativa and Its Application in High Productivity of Germacrene A in Escherichia coli
Source: Front Plant Sci. 2022 Aug 11;13:932966. doi: 10.3389/fpls.2022.932966 (PMC9403833; doi:10.3389/fpls.2022.932966)
Supplement: Supplementary file 1 [file Data_Sheet_1.ZIP › Supplementary figures/Supplementary Figures legend-0611.docx]

[Supplementary](https://www.frontiersin.org/articles/10.3389/fpls.2021.774943/full#S9) Figure 1 The pH effect on LTC2.

[Supplementary](https://www.frontiersin.org/articles/10.3389/fpls.2021.774943/full#S9) Figure 2 SDS–PAGE analysis of the expression levels of LTC2 and various GASs. Lane M, molecular markers; S, the supernatant of the whole cell lysates; IF, insoluble fractions of the whole cell lysates.

[Supplementary](https://www.frontiersin.org/articles/10.3389/fpls.2021.774943/full#S9) Figure 3 SDS–PAGE analysis of the expression levels of LTC2 WT and mutants. Lane M, molecular markers; S, the supernatant of the whole cell lysates; IF, insoluble fractions of the whole cell lysates.

[Supplementary](https://www.frontiersin.org/articles/10.3389/fpls.2021.774943/full#S9) Figure 4 SDS–PAGE analysis of the expression levels of LTC2 WT and mutants. Lane M, molecular markers; S, the supernatant of the whole cell lysates; IF, insoluble fractions of the whole cell lysates.

[Supplementary](https://www.frontiersin.org/articles/10.3389/fpls.2021.774943/full#S9) Figure 5 SDS–PAGE analysis of the expression levels of LTC2 WT and double mutants. Lane M, molecular markers; S, the supernatant of the whole cell lysates; IF, insoluble fractions of the whole cell lysates.

[Supplementary](https://www.frontiersin.org/articles/10.3389/fpls.2021.774943/full#S9) Figure 6 The titer of β-elemene in *E. coli* BL21 star(DE3) carrying pMM and LTC2 triple mutants.
