# Supplementary figures and images for: Protein Engineering of a Germacrene A Synthase From Lactuca sativa and Its Application in High Productivity of Germacrene A in Escherichia coli
Source: Front Plant Sci. 2022 Aug 11;13:932966. doi: 10.3389/fpls.2022.932966 (PMC9403833; doi:10.3389/fpls.2022.932966)

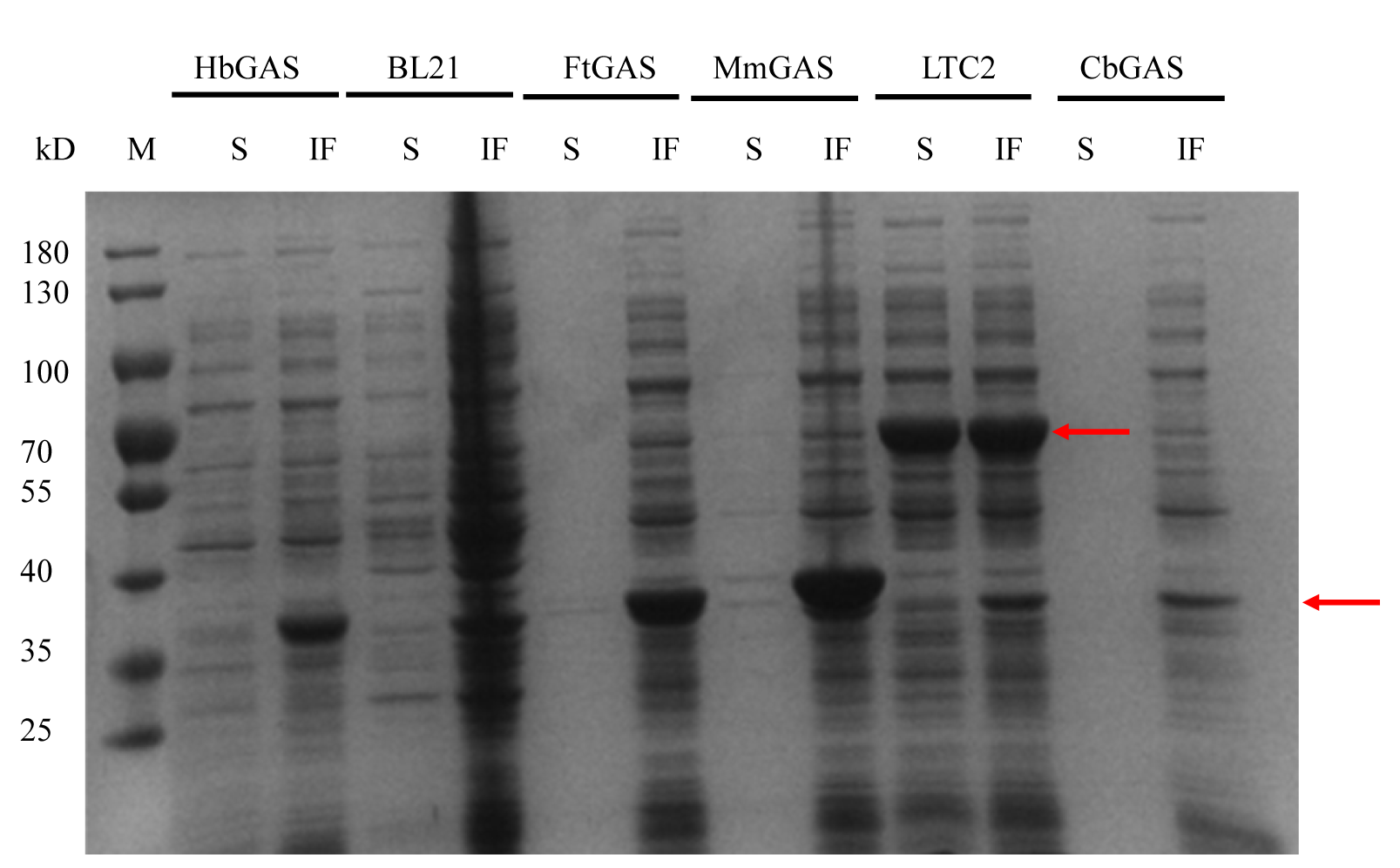

Supplement: Supplementary file 1 [file Data_Sheet_1.ZIP › Supplementary figures/Figure S2-1.tif]

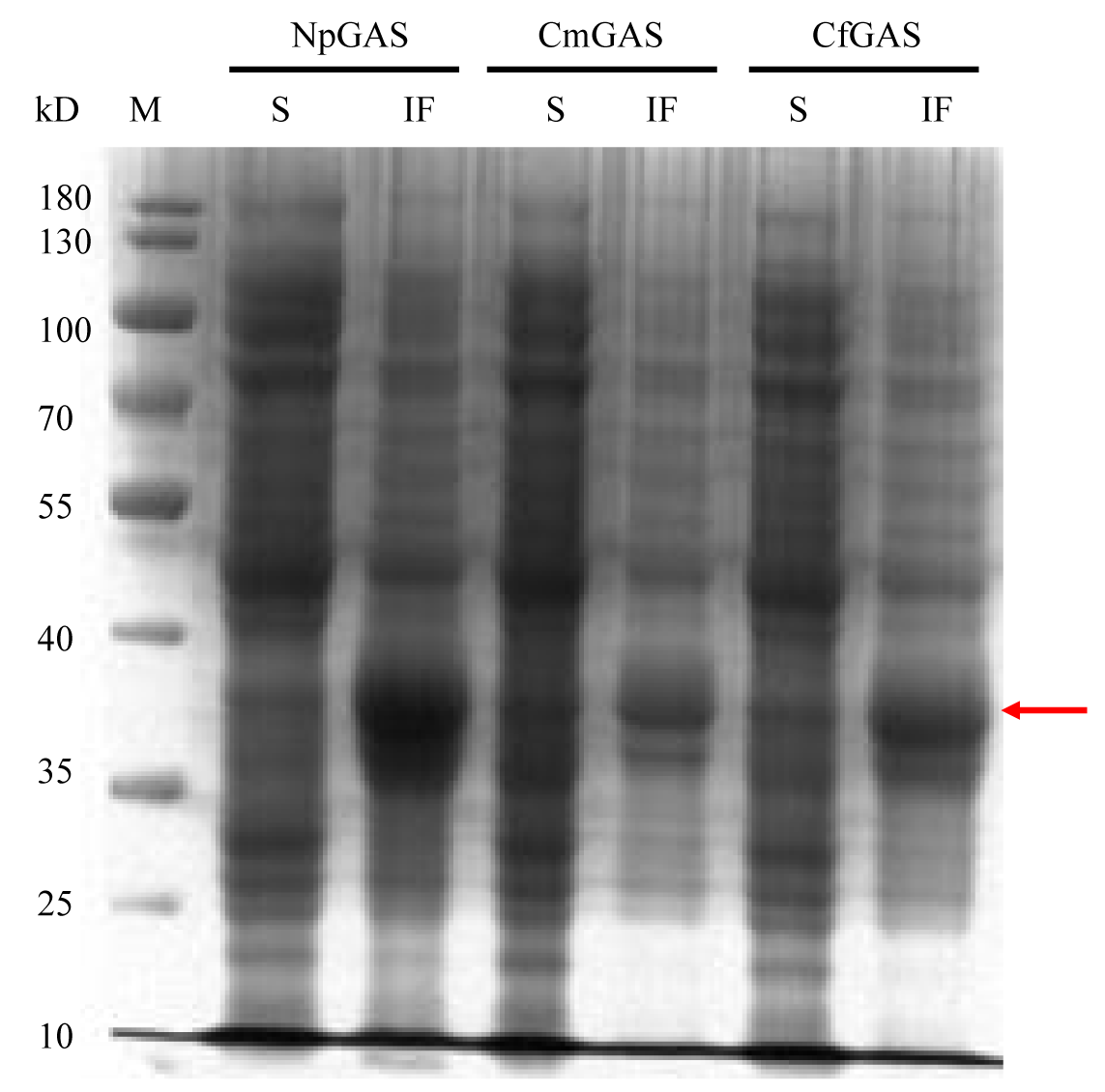

Supplement: Supplementary file 1 [file Data_Sheet_1.ZIP › Supplementary figures/Figure S2-2.tif]

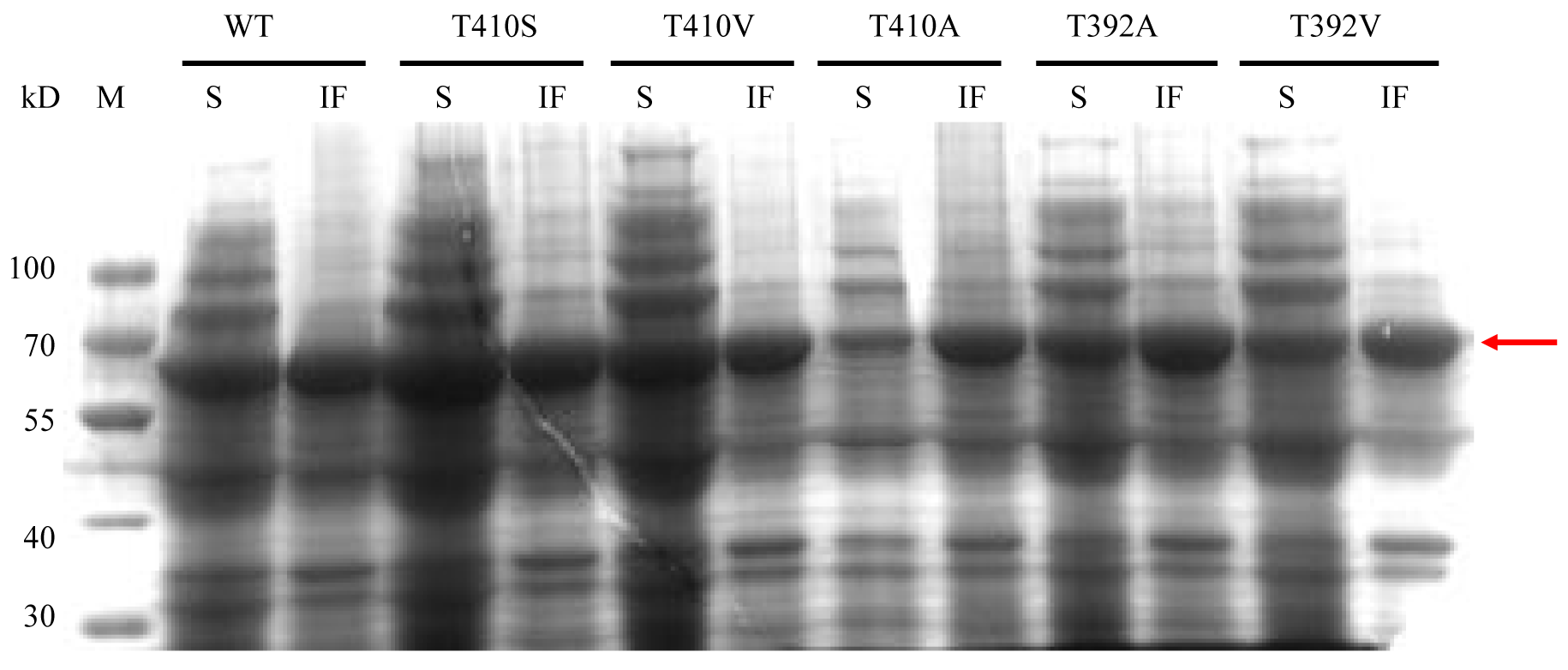

Supplement: Supplementary file 1 [file Data_Sheet_1.ZIP › Supplementary figures/Figure S3.tif]

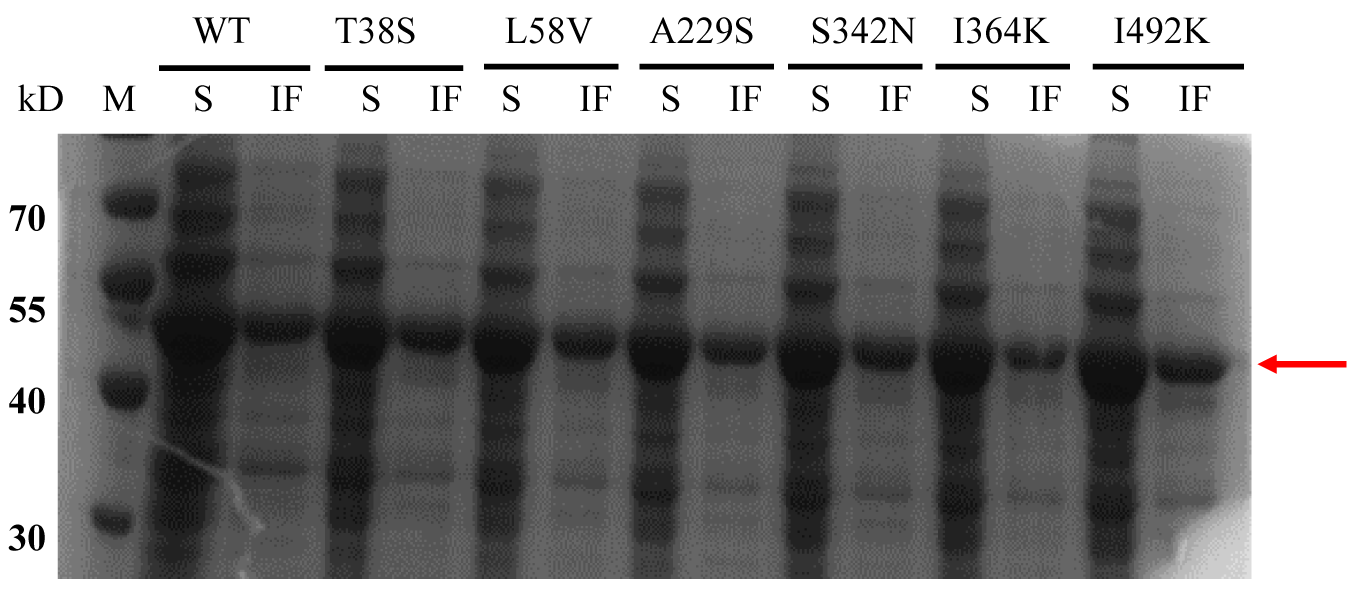

Supplement: Supplementary file 1 [file Data_Sheet_1.ZIP › Supplementary figures/Figure S4.tif]

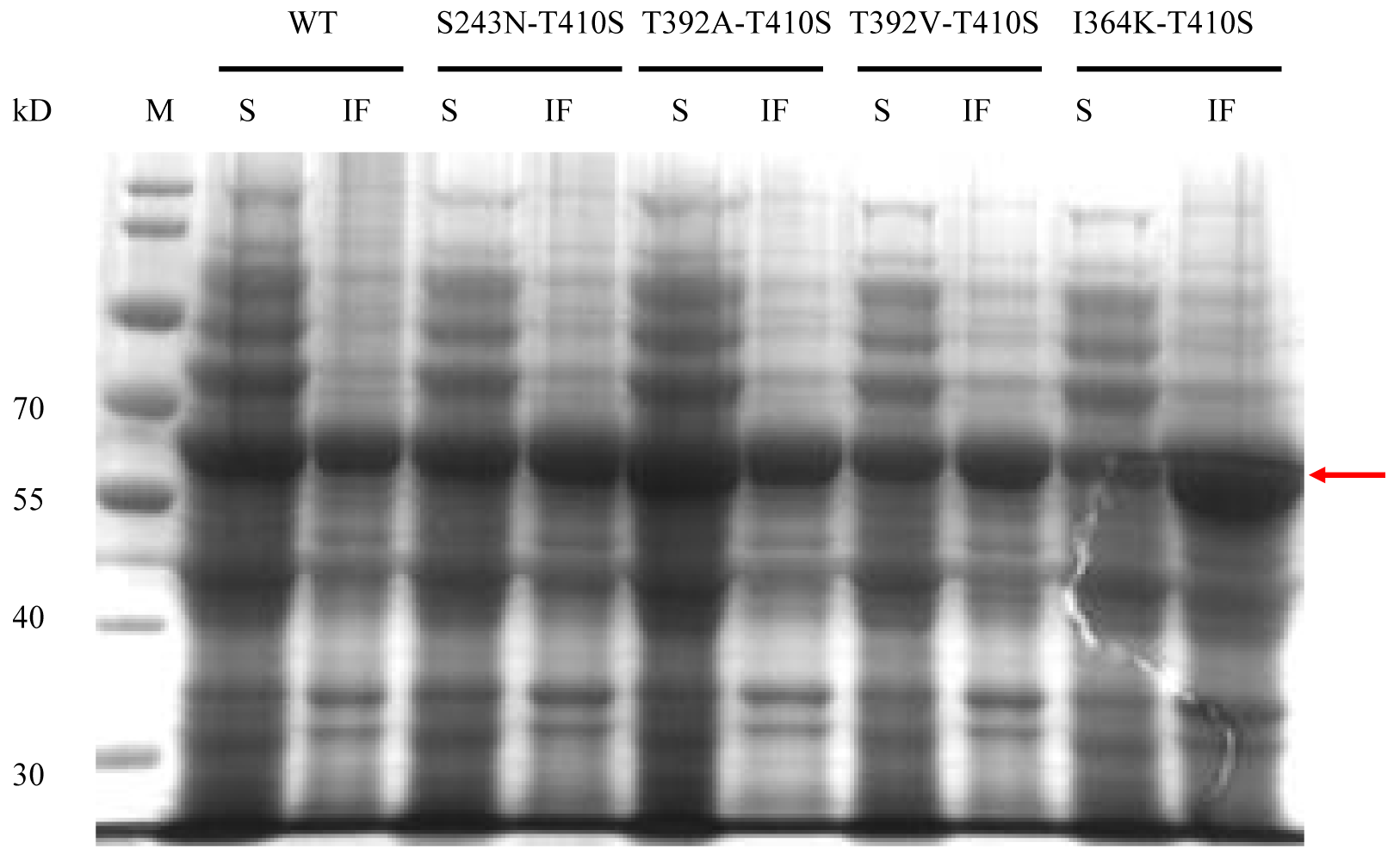

Supplement: Supplementary file 1 [file Data_Sheet_1.ZIP › Supplementary figures/Figure S5 (2).tif]

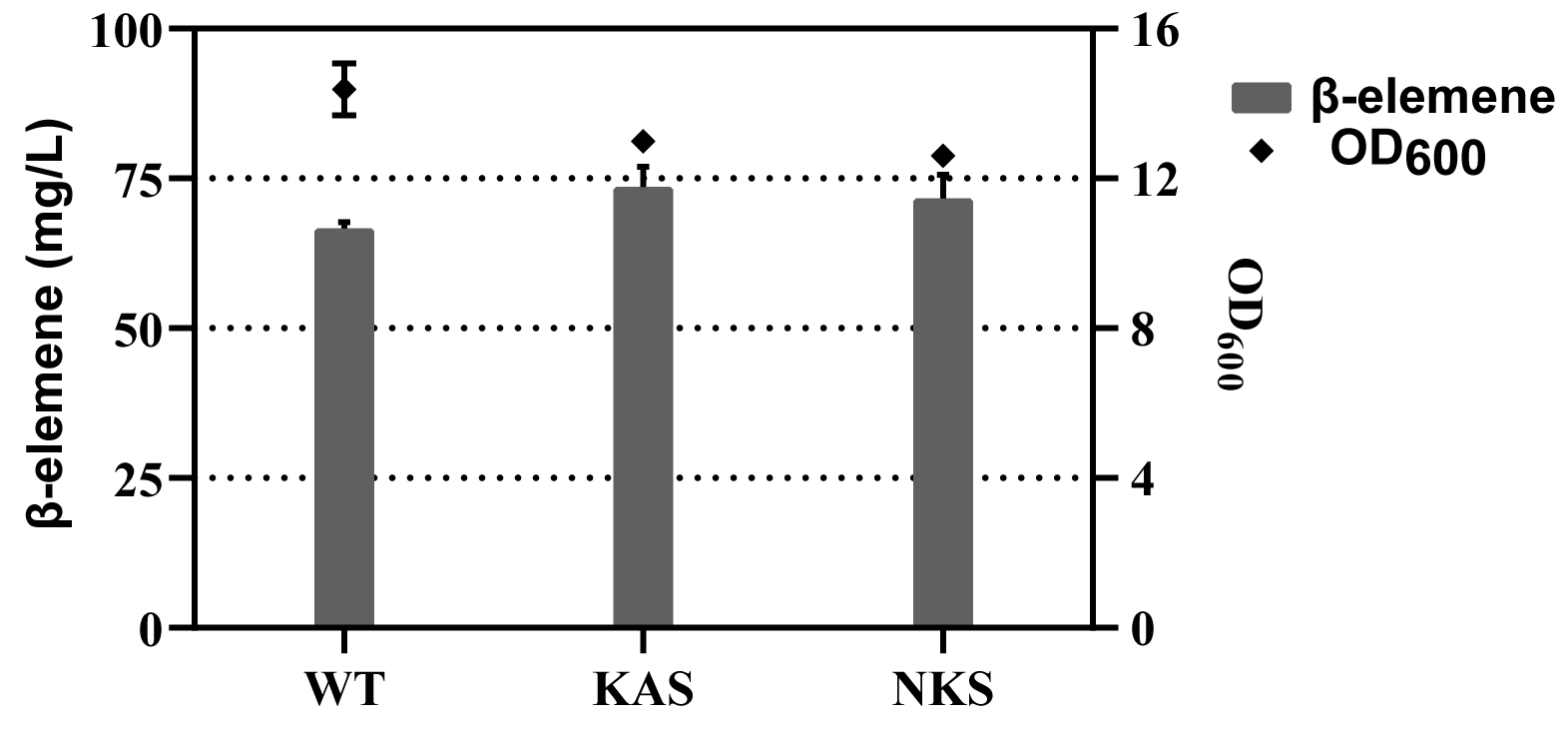

Supplement: Supplementary file 1 [file Data_Sheet_1.ZIP › Supplementary figures/Figure S6.tif]
